# Establishing HIV transmission pathways in Bhutan: a modelling study

**DOI:** 10.1016/j.lansea.2025.100676

**Published:** 2025-09-29

**Authors:** Nisaa Wulan, Lekey Khandu, Debra Ten Brink, Gyambo Sithey, Tashi Dendup, Ye Yu Shwe, Anna Bowring, Nick Scott, Kelvin Burke, Rowan Martin-Hughes

**Affiliations:** a Burnet Institute, Melbourne, Australia; b National HIV, AIDS & STIs Control Program, Ministry of Health, Bhutan; c Centre for Research Initiatives, Thimphu, Bhutan; d Save the Children International, Bhutan Country Office, Thimphu, Bhutan; e UNAIDS Cambodia, Lao PDR; f UNAIDS Cambodia, Malaysia

**Keywords:** HIV transmission, HIV epidemiology, HIV infection, Key population, Bhutan

## Abstract

**Background:**

There are limited and conflicting data regarding HIV transmission and behavioural risk factors, particularly among groups with increased risk of exposure to HIV in Bhutan. This study aims to explore comprehensive pathways to HIV infections among key populations in Bhutan.

**Methods:**

Demographic, epidemiological, and behavioural data were collated to inform an Optima HIV model for Bhutan. The model was calibrated for a period 1990 and 2021 to align with emerging national research into risk attribution of HIV infections and behavioural dynamics of key populations. This was supplemented by qualitative feedback from stakeholder consultations throughout January–June 2022, while maintaining the consistency of the country-accepted output from the 2022 Estimation and Projection Package (EPP-Spectrum model) across all years.

**Findings:**

In 2021, sex work was directly associated with 54% of new HIV infections. In total, 86% of new HIV infections were estimated to be among key and vulnerable populations, their direct partners, and their children. HIV prevalence remained low, ranging from 0.7% to 3.1% among key populations. Due to the relatively short duration of risk activity (average of three years among female sex workers [FSW]), only an estimated 9.7% of undiagnosed people living with HIV could be reached through interventions focused on key populations.

**Interpretation:**

Greater efforts in developing strategies that can prevent new HIV infections among individuals currently at risk—and identifying undiagnosed HIV infections among those with historic risk who are not currently accessing HIV services—could help achieve the elimination of HIV transmission in Bhutan.

**Funding:**

This analysis was funded through The Sustainability of HIV Services for Key Populations in Southeast Asia (SKPA-1) project, funded by the Global Fund to Fight AIDS, Tuberculosis and Malaria under agreement QMZ-H-AFAO, with Health Equity Matters as Principal Recipient. Save the Children is the sub-recipient of SKPA in Bhutan.

**Research in context**

**Evidence before this study**

We searched PubMed for English-language studies published up to April 8, 2025 using the terms “(HIV transmission) OR (HIV infection) OR (key population) OR (drayang) AND (Bhutan)”, resulting in 115 titles. A total of 8 studies has noted sources of HIV transmission or characteristic of specific key populations including sex workers. To date, there have been no studies describing the overall HIV transmission pathways focusing on the partnerships, risk behaviour, and risk transitions between key population and non-key population groups.

**Added value of this study**

We conducted a modelling analysis, calibrated a detailed dynamic epidemic model to all available cross-sectional data, and established comprehensive pathways to HIV infections in Bhutan. Focusing on the specific context of Bhutan, it highlights the importance of understanding the challenges and strategies towards reaching the fast-track targets ending the HIV epidemic through increasing coverage of HIV prevention and testing for key populations associated with sex work. This could lead to reducing barriers to access to HIV services particularly for those who are not easily reachable through existing prevention interventions.

**Implications of all the available evidence**

The HIV epidemic in Bhutan that is concentrated among key populations highlights the need for targeted interventions tailored to meet the needs of the affected populations. Efforts to design HIV interventions that tailor specific context and behaviours could lead to elimination of local HIV transmission.

## Introduction

Bhutan has maintained a low-level HIV epidemic over the last decade, with annual new HIV infections estimated at less than 100 since 2011.^1^ The estimated number of people living with HIV was 1300 in 2023, with an estimated 56% of people living with HIV diagnosed, 98% of those diagnosed receiving antiretroviral therapy, and 84% of those on treatment being virally suppressed.^2^

Established in 1988, the National HIV, AIDS & STIs Control Program (NACP) provides leadership to the implementation of the National HIV, AIDS and STI Prevention and Control Strategic Plan, prioritizing key populations including female sex workers (FSW) and their clients, men who have sex with men (MSM), transgender women (TGW), and people who inject drugs (PWID).^3^^,^^4^ Within the strategic plan, women working at or visiting entertainment establishments (e.g., drayangs or traditional entertainment dance bars, karaoke clubs, disco bars) where high-risk sexual behaviours are frequently initiated, such as sex work and transactional sex, are also categorized as one of key affected population groups—hereinafter referred to as high-risk women (HRW) consistent with national terminology.^5^ Other groups such as migrant workers, mobile populations, people in monastic institutions, and uniformed personnel are also recognized as being vulnerable to HIV.^3^^,^^4^^,^^6^

The enabling legal and policy environment in Bhutan is evolving. In 2021, Bhutan decriminalized consensual same–sex relations by amending provisions of the Penal Code that had previously criminalized ‘unnatural sex’.^7^ However, sex work remains criminalized under the Penal Code, including promotion of prostitution (e.g., brothel keeping and soliciting).^8^ Similarly, drug use, including injecting drug use, is criminalized under the Narcotic Drugs, Psychotropic Substances and Substance Abuse Act, which prohibits possession and consumption of controlled substances.^9^

HIV prevention services for key populations are available through civil society and community-based organizations, providing a package of HIV prevention interventions including community outreach activities, distributing condoms, promoting safe sex, assisting in referrals for testing and providing psychosocial support.^10^ Health Information and Service Centers (HISC) provide voluntary counseling and testing services for HIV/STIs, as well as health education and awareness on prevention of HIV and STIs, condom distribution, and outreach-related activities.^6^ However, it has been documented that the key populations have not been easily reachable through traditional prevention interventions such as HIV-testing and counselling services, including facility-based and community-based testing.^5^^,^^11^, ^12^, ^13^, ^14^, ^15^

The implementation of pre-exposure prophylaxis has been phased in gradually since 2024, initially targeted to reach MSM, TGW and FSW delivered through hospitals and HISCs.^16^ HIV treatment and care are guaranteed under Bhutan's constitutional commitment to free basic health services. The 2024 National Guideline on Treatment and Management of HIV and AIDS adopted same-day initiation of antiretroviral therapy, transition to dolutegravir-based regimens, and multi-month dispensing.^3^^,^^17^

There have been limited data on the HIV transmission dynamics in Bhutan, as outlined by Tshering et al. in a comprehensive overview of the HIV epidemic in 2016.^18^ In response to this, since 2019, the NACP implemented a case-based surveillance system to assess detailed HIV exposure risks among individuals with newly diagnosed HIV that can be analyzed over time.^19^ A 2021 retrospective study analyzing HIV cases from 1993 to 2021 suggested that multiple heterosexual partnerships involving sex workers, clients of sex workers and their spouses were the predominant sources of transmission.^20^ This retrospective survey was contrary to 2021 HIV sentinel surveillance (HSS) study findings of few HIV infections among sex workers, with an estimated HIV prevalence among sex workers of <0.1% (95% CI 0.0%–3.9%),^21^ leading to considerable uncertainty around HIV transmission dynamics in Bhutan and the appropriate government response. The HSS study conducted HIV testing through peer-driven recruitment sampling and identified zero HIV infections; however, the sample included a mix of FSWs and HRWs. Surveys of entertainment workers in drayangs indicated a short average working duration of less than 8 months in these venues,^13^ suggesting that the short duration at risk of HIV transmission could be one possible explanation for the difference between the HIV surveillance data and risk attribution survey.

Data on prevalence of HIV risk behaviours among MSM and TGW was scarce before 2016, with only small-scale studies conducted.^22^^,^^23^ The HSS study conducted in 2021 found the HIV prevalence among samples of MSM and TGW to be 0.9% and 0.0%,^21^ respectively. Despite these low estimates, a high proportion of TGW (59%) reported engaging in commercial sex work with an average of 5.4 sex partners in the last 30 days. In the same study, a smaller proportion of MSM reported engaging in commercial sex (8%) with an average of 1.8 sexual partners in the last 30 days. This suggests that while current HIV prevalence may be low, there remains potential for the epidemic to expand among MSM and TGW.

Bhutan utilized the EPP-Spectrum model^24^ to prepare official national estimates of key HIV indicators to assess overall epidemic trends from 1990 to 2021. The development of the Spectrum model has been supported by the Joint United Nations Programme on HIV and AIDS (UNAIDS) Reference Group on Estimates, Modelling and Projections. It has been applied to over 100 countries to produce estimates of key HIV indicators using their latest behavioural and HIV surveillance data, survey, and program data, combined with demographic data.^25^ Spectrum also calculates uncertainty around the model estimates employing the Monte Carlo techniques involving, by default, 300 iterations and randomly selecting parameter values for each of indicators for each iteration.^25^ However, this modelling does not provide key population specific HIV estimates or detailed epidemic dynamics.

Therefore, despite the time trends of key HIV indicators and nation-wide studies into HIV transmission, gaps remain in understanding the dynamics of HIV epidemics in Bhutan. There have been no studies previously conducted to determine comprehensive pathways to HIV infections among key population in Bhutan utilizing emerging national surveys.

Epidemiological modelling is a useful tool for analyzing HIV data from multiple sources to quantify the dynamics of HIV epidemics accurately and reliably.^26^ It also allows establishing the population-level transmission pathways beyond the observed data to guide policy makers in improving HIV/AIDS response. This study uses findings from a 2022–23 Optima HIV modelling analysis aimed to calibrate a detailed dynamic epidemic model to all available cross-sectional data to paint a holistic picture of the HIV epidemic, and model comprehensive pathways to HIV infections and modes of transmission. Given a low-level HIV epidemic in Bhutan, maintaining such a low-level HIV epidemic in a small country may come with both unique challenges in case finding as well as opportunities to achieve ambitious targets toward eliminating local HIV transmission.

## Methods

### Study setting

Bhutan is situated in the Himalayas between India and China and had an estimated population of 775,000 people in 2023. Reported HIV cases in Bhutan are low both in absolute numbers (cumulatively 795 diagnosed by the end of 2022)^27^ and relative to population size, with estimated HIV incidence of <0.1 per 1000 uninfected people.^2^ The Optima HIV study into HIV transmission pathways was undertaken from January 2022 to June 2022.

### Model development

**Fig. 1:**
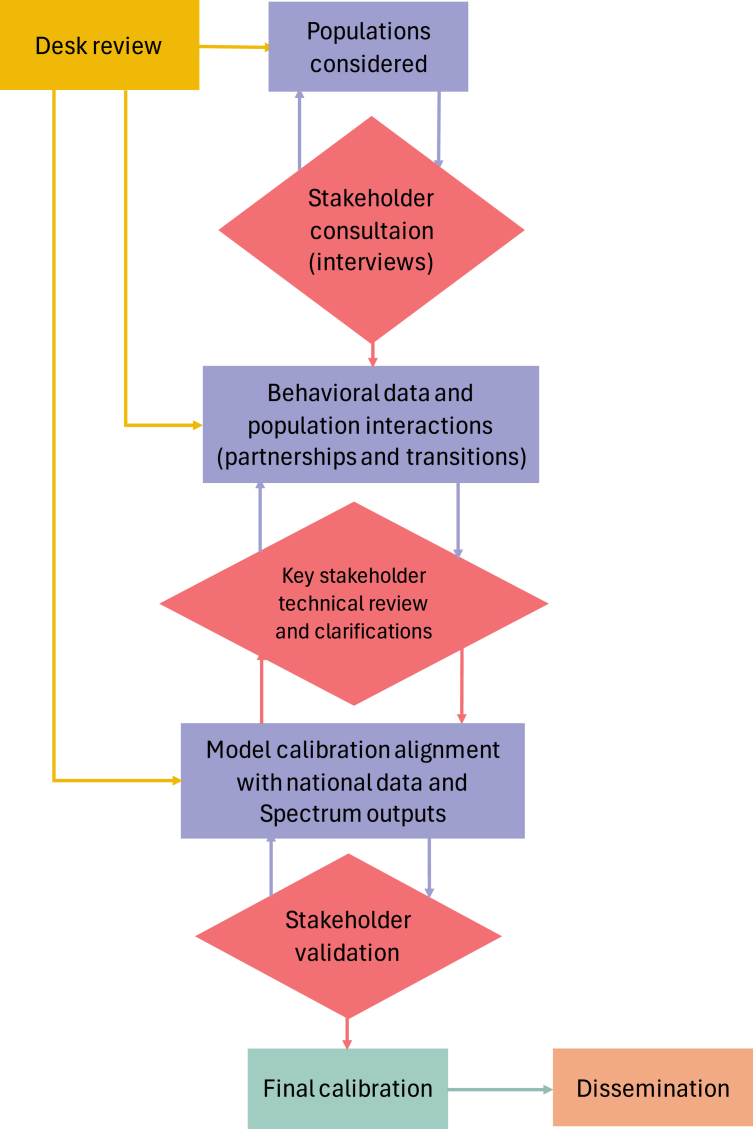
Schematic diagram for process of model development and validation.

The study involved stages as illustrated in Fig. 1, to strengthen empirical evidence of HIV transmission dynamics in Bhutan. This approach captured published evidence on the HIV epidemic in Bhutan, underpinned by a scoping review of previous studies and surveys. Simultaneously, the country stakeholders led in data collation for model inputs, validation of model calibration, and ultimately the robustness of model outcomes. Each of the model development stages are described below.

### Desk review

The study process commenced with desk review to identify publicly available data sources between 2010 and 2022 including surveys and peer-reviewed articles. Information gathered from desk review was summarized to identify the previously defined key populations and their risk behaviours used in research or country-level surveys, knowledge gaps around primary routes of HIV transmission, and what has been done in maintaining a low-level HIV epidemic.

### Stakeholder consultation

Stakeholders from NACP, Save the Children, key population organizations–namely Lhak-Sam, Pride Bhutan, Red Purse Network, and Chithuen Phendhey Association, and key population representatives participated in stakeholder consultations between January and June 2022. Stakeholder consultations played a key role in understanding the country's HIV situation, identifying and defining relevant key populations, mapping out the available country-level surveys/data, and validating model inputs and preliminary findings. For example, stakeholder inputs resulted in adjustments in the duration of high-risk periods of HRW and risk transitions between population groups (HRW and FSW, as well as MWUD and clients of FSW) to capture overlapping risks. A complete list of stakeholder consultations is available in Appendix A.

### Study population

Key populations modelled were FSW, HRW, MSM, TGW, and men who use drugs including alcohol (MWUD) based on stakeholder input and adequate evidence, behavioural data, and population size estimates to generate modelling inputs. To date, there have been no published studies to suggest that the HIV epidemic is initiated and expanded among PWID in Bhutan, nor is there an official estimation of the size of PWID. However, stakeholders expressed growing concerns about potential links between high levels of alcohol and substance use and HIV transmission, as well as historic injecting drug use.

Previous studies indicated a substantial proportion of substance use among adolescents (alcohol and illicit drug use at 24% and 12%, respectively) in Bhutan.^28^ The non-communicable disease risk factors of Bhutan STEPS survey 2019 found that over 51% of current drinkers engage in heavy episodic drinking, defined as the consumption of six or more standard drinks on a single occasion, at least once in the last 30 days.^29^ Considering the potential role of substance use in the spread of HIV/STI infections^30^^,^^31^ along with stakeholder concerns, MWUD were included in the analysis. MWUD were modelled as a single population including an assumed 10% of PWID due to insufficient data to disaggregate by specific substance. Stakeholders considered MWUD to be an important population group to model due to mixed and uncertain risks. In addition, they were considered an accessible population group for potential HIV interventions, if the need were established, given engagement with other social support outreach programs that are not funded through the HIV program.

The definitions of key populations included in this analysis are presented in Table 1. Non-key populations were disaggregated by sex and age resulting in female and male population groups aged 0–14, 15–19, 20–24, 25–49, and 50 years and older.

**Table 1:** Key population groups modelled in this analysis.

Population group	Definition
FSW	Biological females, 15–49 years old, who have had commercial/transactional sex in the last 12 months.
HRW	Biological females, 15–49 years old, who work at or visit hotspots (e.g., dance bars or drayangs, karaoke clubs, disco bars) defined as environments where high-risk sexual behaviours are frequently initiated (e.g., commercial sex networking within and between key populations).
Clients of female sex workers (clients of FSW)	Clients of female sex workers, who have paid money or goods in exchange for sex in the last 12 months.
MSM	Biological males, 15–49 years old, who have had anal sex with another male in the last 12 months including those who find and meet male sex partners through online applications.
MWUD	Biological males, 15–49 years old, who have used illicit drugs including use of cannabis, ‘brown sugar’ heroin, solvent/glue (sniffing), pharmaceuticals for pleasure and injecting, and harmful use of alcohol. Harmful use of alcohol was considered based on the national definition of heavy episodic drinking, defined as consuming 6+ standard drinks on at least one single occasion within the last 30 days.
Transgender women (TGW)	People who were assigned male at birth, 15–49 years old.

FSW: female sex workers; HRW: high-risk women; MWUD: men who use drugs including alcohol; TGW: transgender women.

### Model design

The analysis was conducted using Optima HIV, a population-based compartmental model of HIV transmission, and disease and care cascade progression (disaggregated by age, sex, and risk factors). The Optima HIV allows for quantification of HIV incidence, prevalence, and attributable fractions of new HIV infections among key populations informed by various data sources and tailored to national contexts.^32^ A detailed description of Optima HIV can be found in Kerr et al.^26^ Description of model parameters incorporating behavioural risks affecting HIV transmission, underlying progression of HIV and treatment recovery including additional population specific parameters to capture risk differences between populations are reported in Appendix B.

### Model inputs

Demographic, epidemiological, and behavioural data that were not publicly accessible were supplied by the country team and collated for the inputs to the Optima HIV model for Bhutan. This compilation included data derived from integrated biological and behavioural surveillance surveys,^15^ national annual health reports,^1^^,^^27^ and other relevant reviews or assessments of HIV services. The estimation of the size of key populations was informed by the most recent national key population size estimation survey,^5^ which was conducted between November 2019 and January 2020.^5^ Detailed model inputs associated with populations, sexual partnerships and condom use are presented in Appendix B.

### Model calibration

After including model inputs, key parameters were iteratively calibrated through a consultative process, to ensure model consistency with (a) the 2021 study on the risk attribution on sources of HIV transmission,^20^ (b) the 2021 HSS prevalence study,^21^ and (c) overall trends in annual HIV prevalence, incidence, and mortality from 1990 to 2021 in the country-accepted output from the 2022 EPP-Spectrum model.^24^ These were used to compare with the Optima model outputs. Each study categorized populations differently to fit their measurements and purposes, so we interpreted source population groups to best reflect key populations modelled in this study (Table 2) with input from stakeholders to ensure the alignment of programmatic data and operational research.

**Table 2:** Proportion of risk factors aligned with against the modes of transmission based on risk attribution reported in the HIV modes of transmission 2021 retrospective study.^20^

Risk factors aligned with the modes of transmission	Optima population group	Percentage of HIV infection reported in the HIV modes of transmission 2021 retrospective study
Sex workers	FSW	13%
Heterosexual contact with sex workers/clients of sex workers	Clients of FSW, HRW	41%
Heterosexual contact with HIV with a person injecting drug use	MWUD	<1%
Male-to-male sexual contacts	MSM/TGW	2%
Vertical transmission	Children (0–14)	9%
Other heterosexual contacts	Non-key population adults (15+)	35%

FSW: female sex workers; HRW: high-risk women; MWUD: men who use drugs including alcohol; TGW: transgender women.

Model inputs and calibration were first reviewed and updated through a series of ten online workshops between the core technical group including authors to ensure consistency with: emerging national data and suitability for answering questions aligned with national priorities (National HIV/AIDS and STIs Control Program); model structure (Burnet Institute); community-based organization perspective (Save the Children); regional trends and prior national modelling (UNAIDS). A stakeholder consultation workshop including all previously consulted stakeholders was held in May 2022 to review preliminary findings. Final model calibration was validated by the Bhutan Ministry of Health, core technical group, and key population representatives in June 2022 (Appendix A). Further detail on model calibration is described in Appendix B.

### Modelling outcomes

The calibrated model was then used to generate additional insight into overall HIV transmission dynamics, and the implications for identifying undiagnosed people living with HIV. While people with a history of being in a key population were not modelled separately, we estimated the proportion of each population (including the proportion of people living with HIV) with historic risk which could be tracked over time based on population group transitions in the model (e.g., beginning or ceasing entertainment work). The results below have been structured to follow the modelling process.

### Ethics approval

Ethical approval was not applicable.

### Role of the funding source

Health Equity Matters contributed to the study design and coordination. The funders had no role in data collection and analysis, decision to publish, or preparation of the manuscript.

## Results

**Fig. 2:**
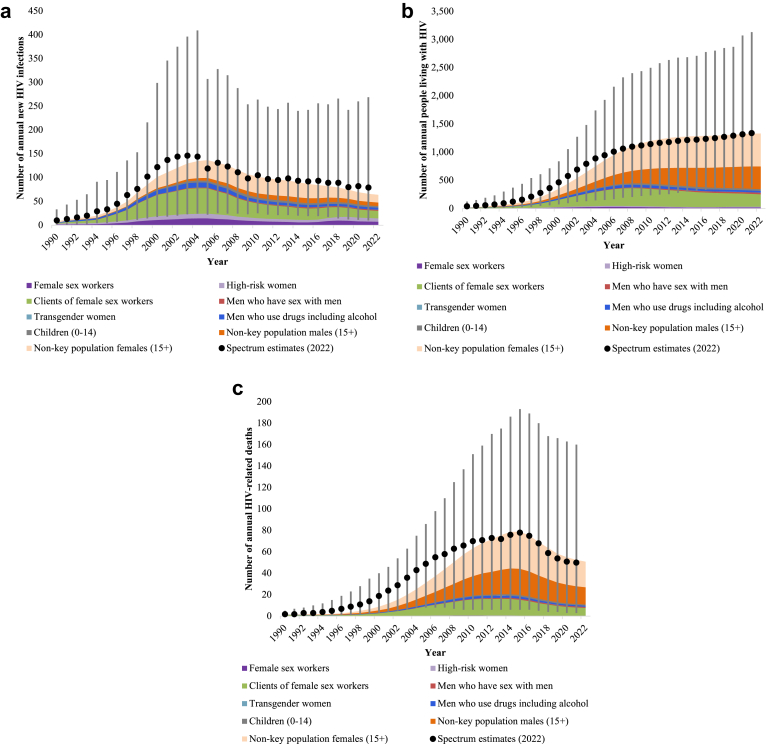
Estimated new HIV infections, people living with HIV, and HIV-related deaths from 1990 to 2022. EPP-Spectrum uncertainty bounds are visualized vertically against the Optima HIV projection over time. a. Estimated new HIV infections from 1990 to 2022. b. Estimated people living with HIV from 1990 to 2022. c. Estimated HIV-related deaths from 1990 to 2022.

Fig. 2 shows distinct trends and patterns across various population groups, each contributing to the overall HIV epidemiological landscape. The Optima HIV baseline model aligned with EPP-Spectrum estimates of a rising trend of new HIV infections from the early 1990s to mid-2000s peaking between 2003 and 2005 and steadily decreasing to approximately 66 new HIV infections in 2021 (Fig. 2a). Fig. 2b shows a gradual increase in people living with HIV over the years reaching an estimated 1328 in 2021 while HIV-related deaths have been reducing to an estimated 53 in 2021 (Fig. 2c) since 2015. The uncertainty ranges for annual number of new HIV infections, people living with HIV, and HIV-related deaths are available in Appendix C.

Within these trends, Optima HIV estimated that initially, key populations such as FSW and their clients experienced sharp increases in new infections, peaking around the mid-2000s, followed by significant declines. New infections among MSM are estimated to have remained low at less than 3 on average per year in Bhutan. New infections occurring through non-key population males and females showed a gradual increase until the mid-2000s before also declining. New infections among children, primarily through vertical transmission, peaked in the early 2000s but then rapidly decreased in response to scaling up of interventions to prevent vertical transmission from mother to infant.

**Fig. 3:**
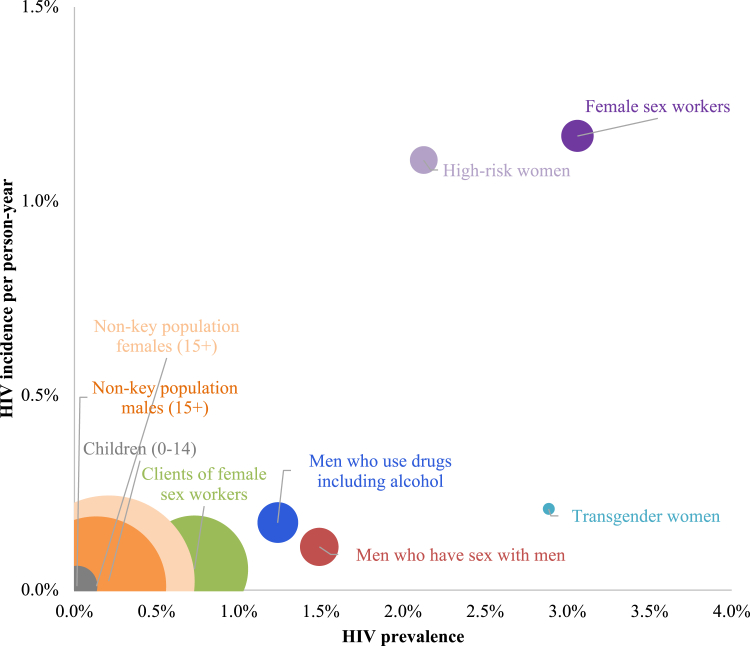
Overview of modelled HIV prevalence, modelled HIV incidence, and total number of people living with HIV (bubble size) in 2021. Estimated people living with HIV in 2021—Female sex workers: 20; High-risk women: 14; Clients of female sex workers: 224; Men who have sex with men: 28; Men who use drugs including alcohol: 31; Transgender women: 2; Children: 32; Non-key population females: 581; Non-key population males: 394.

The calibrated Optima HIV model was consistent with HIV prevalence by key population from the 2021 HSS study (Table 3). The Optima HIV model included further disaggregation of key populations relative to the 2021 HSS study such as clients of FSW and HRW. For key populations where survey data were not available, Optima HIV estimated the prevalence of HIV among clients of FSW, HRW and MWUD to be 0.7%, 2.1%, and 1.2% respectively. Fig. 3 shows modelled HIV prevalence estimates ranged from 0.7% to 3.1% among key populations and 0.02% to 0.26% among non-key population groups. Modelled HIV incidence ranged from a low of 0.002 per 100 person-years among children to a high of 1.2 per 100 person-years among FSW. The bubble size represents the estimated total number of people living with HIV. Non-key population females and males have the highest estimates for people living with HIV, despite the lowest HIV prevalence and incidence estimates.

**Table 3:** HIV prevalence estimates by key population, comparing Optima outputs to the 2021 HSS study.^21^

	Optima HIV modelled prevalence 2021 (95% CI)	HSS study HIV prevalence 2021 (95% CI)
FSW	3.1% (1.8%–6.4%)	0.0% (0.0%–3.9%)
Clients of FSW	0.7% (0.5%–1.3%)	NA
HRW	2.1% (1.2%–4.3%)	NA
MSM	1.5% (0.8%–2.8%)	0.9% (0.0%–2.6%)
MWUD	1.2% (0.8%–2.2%)	NA
TGW	2.9% (1.5%–5.1%)	0.0% (0.0%–4.0%)

FSW: female sex workers; HRW: high-risk women; HSS: HIV sentinel surveillance; MWUD: men who use drugs including alcohol; TGW: transgender women.

**Fig. 4:**
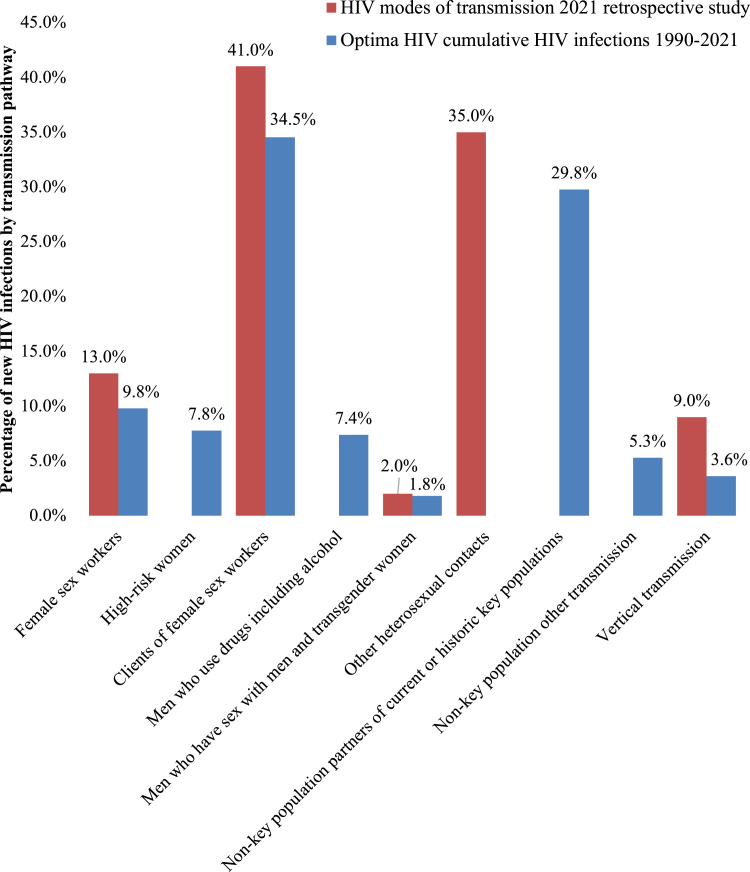
Modes of transmission 2021 retrospective cohort analysis compared with Optima HIV model cumulative infections 1990 to 2021.

Cumulative incidence outputs of the calibrated Optima HIV model from 1990 to 2021 were consistent with sources of HIV transmission by key population from the 2021 retrospective study of diagnosed people living with HIV, subject to additional detail in modelled key populations (Fig. 4). This highlights which key populations were most affected in Bhutan historically. It is likely that the 2021 retrospective study classified MWUD with clients of FSW together due to overlapping dynamics of higher risk sexual and drug use behaviours in both clients of FSW and MWUD.

In 2021, Optima HIV estimated approximately 54% (36/66) of new infections were acquired through heterosexual transmission among FSW, HRW, clients of FSW, and MWUD. Other heterosexual transmission consisting of non-key population adults, regular female partners of clients of FSW, and male partners of previously HRW contributed 37% (25/66) of new infections. About 3% (2/66) and 5% (3/66) of new infections occurred through male-to-male sexual transmission including TGW, and vertical transmission, respectively.

Both FSW and HRW were reported to be working in drayangs and other entertainment venues and experienced risk of HIV infection through multiple sexual partnerships. Survey data among drayang girls (including both FSW and HRW) indicated a mean working duration of 8.4 months.^13^ However, this survey only captured people still working in entertainment venues. Stakeholder consultations (Appendix A) indicated that this was also likely an underestimate of the overall rates of transition due to (a) seasonal work for some young women, including moving to work in entertainment venues in different regions of the country, and (b) especially likely to under-estimate the duration of sex work for home-based FSW, who were not included in the survey. The modelled mean durations before returning to non-key populations were based on stakeholder feedback and model calibration to be 1.5 years for HRW and 3 years for FSW, recognizing that some FSW have far longer durations of sex work. Based on survey data,^13^ the annualized transition rate between HRW and FSW was set to 25%, with approximately 70% of FSW and HRW being aged 18–24 and the remainder 25–49.

Clients of FSW and MWUD are characterized by overlapping patterns of HIV risk behaviours, specifically engaging in buying sex and substance use. Approximately 14% of urban males aged 15–49 in Bhutan were estimated to be engaging in transactional sex which informed the population size estimate for this population.^33^ However, specific surveyed populations including migrant workers, police, military, truck drivers, and taxi drivers, all had much higher rates ranging from 21% to 66% depending on profession and location. The mean durations in these professions for those surveyed while still in those professions ranged from 4.8 to 9.6 years, implying a mean duration of closer to double those values overall. Stakeholder consultation and model calibration informed the modelled mean duration of 11 years before returning to non-key populations for both clients of FSW and MWUD. Based on the overlapping risks and survey data,^33^ the annualized transition rate between MWUD and clients of FSW was set to 10% of the MWUD population (approximately 1% of the clients of FSW population). A higher proportion of clients of FSW (44%) and MWUD (36%) were assumed to be males aged 20–24 relative to the national population (24% of adult males in 2020), consistent with the identified male populations with higher rates of commercial partnerships.

Although over a third of new infections were estimated to occur among non-key populations, among non-key population females aged 15+ more than 85% of new HIV infections could be attributed to partnerships with males who were in key populations or who had been in the past. Among non-key population males aged 15+, approximately 40% of new HIV infections could be attributed to partnerships with females who were in key populations or who had been in the past. In 2021, 57 (86%) of the estimated 66 new HIV infections occurred among key populations or were linked to partnerships with individuals who were currently or previously part of key populations. Cumulatively from 1990 to 2021, the proportion was even higher, at 2390 (93%) of 2570 cumulative HIV infections.

**Fig. 5:**
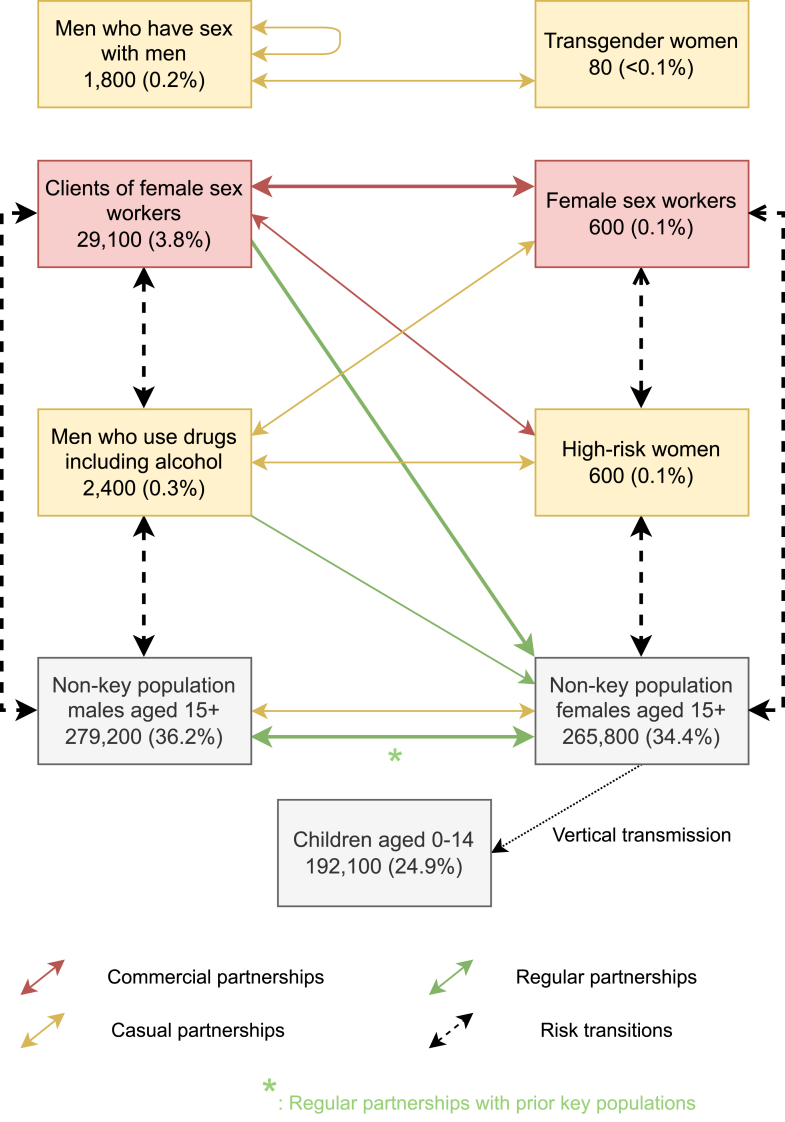
Key modelled pathways to HIV infection. Thicker lines highlight primary routes of modelled transmission.

The calibrated dynamic epidemic model gives insights into the primary routes of HIV transmission through these partnerships and risk transitions between key population and non-key population groups (Fig. 5). These risk transitions between key and non-key population groups resulted in HIV prevalence within non-key population groups despite low levels of transmission through either casual or commercial partnerships. Although people with a history of being in a key population were not modelled separately, it could be inferred through the model calibration that the non-key population males and females who were at a higher risk of HIV infections included regular female partners of current and historic clients of FSW and MWUD, and regular male partners of historic FSW and HRW.

## Discussion

This Optima HIV analysis, which incorporated both historical and recent HIV epidemiological data, aligned with observed data and corroborated the low prevalence of HIV among FSW and other key populations, resulting in a consistently low positive yield of HIV testing among key populations. Notably, most new infections were linked to sex work, with a significant proportion of new infections occurring among FSW, clients of FSW and HRW. This study also established comprehensive HIV transmission pathways accounting for the possibilities of infection from commercial, causal or regular partnerships alongside tracking the historic risk over time-based on population group transitions.

FSWs were estimated to work for an average of approximately three years with elevated vulnerability to HIV/STI infection due to their engagement in commercial sex with multiple clients. However, there is substantial variation in both the duration of sex work and the level of risk within this population. Those employed in the drayangs generally earned higher incomes and consequently spent shorter durations in sex work compared to those experiencing financial hardships,^13^ who were compelled to remain in the profession for longer periods, often exceeding three years. On the other hand, home-based FSWs were likely to have a longer working duration and were not easily reached through outreach programs.^5^ It has been documented in various settings^34^^,^^35^ that HIV and syphilis prevalence are higher among FSW who engage in a longer duration of sexual activity. Outreach targeting home-based FSWs could be planned by considering the feasibility of service uptake,^36^ for example through interventions delivered discreetly in a place approved by FSWs and occurring during off-peak times. Such targeted interventions have now become more important considering the enforcement of the closure of drayangs by the government in 2022, where many drayang workers could be operating from homes.

Similar to FSW, HRW were also at high risk of acquiring HIV through casual sexual partnerships with clients of FSW or MWUD. Stakeholder consultations indicated that HRW who worked in the drayang establishments also frequently moved from one drayang to another depending on seasonal labor demands. Tailoring interventions to specific contexts and behaviours is necessary to effectively reduce the risk of HIV transmission, such as implementing HIV prevention programs directly in high-risk venues and offering vocational training and alternative employment opportunities to reduce financial dependency on occupations at risk of exposure to HIV.^37^

It was estimated that 86% (n = 57) of undiagnosed people living with HIV in Bhutan in 2021 either historically belonged to a key or vulnerable population, including FSW, HRW and clients of FSW, or were a direct partner or child of people belonging to a key population. However, due to the short duration of risk and with no interventions reaching clients of FSW or partners, only 10% of undiagnosed people living with HIV in Bhutan were estimated to belong to an accessible key population. This presents challenges in the implementation of targeted HIV prevention and testing due to a need to reach partners of key populations and individuals who had previously been part of a key or vulnerable population. Combined with operational challenges in reaching longer-term home-based FSW with HIV prevention services reported by stakeholders, it is likely that the prevalence of undiagnosed infections is likely to remain high in the absence of being able to reach those exposed to historic risk. Improvements in strategies to expand HIV prevention programs for key populations complemented by index testing for identifying sexual partners of key populations or individuals with historic risk could provide a way forward. Moreover, stigma and discrimination contributes to barriers to accessing HIV services in Bhutan.^10^^,^^23^ Educational and culturally sensitive interventions in healthcare settings and communities to increase sensitization on stigma and discrimination showed a positive impact in reducing stigma and increasing HIV test uptake.^38^^,^^39^

This study did not find evidence that a substantial proportion of new HIV infections in Bhutan are linked to people who have a primary risk factor of alcohol or other substance use. However, there is very limited data on these populations and surveys of acquisition risk have attributed past infections to injecting drug use. Overlapping patterns of HIV risk behaviours of buying sex and substance use among clients of FSW and MWUD created more complex pathways for HIV infection. Notably, evidence indicates a lower likelihood of condom use among those who have sex under the influence of alcohol compared to those who had sex without being under the influence of alcohol.^13^ Similarly, in comparison with FSWs recruited at venues, those sex workers referred by peers were more likely to have sex under the influence of alcohol (80.1% vs. 51.5%, P < 0.001) and more paying partners.^40^

In Bhutan, presently there is no direct way to reach clients of FSW with HIV services which implies under-diagnosed HIV infections. Undiagnosed clients of FSW and MWUD living with HIV increase the risk of HIV infection for their regular non-key population female partners, FSW, and HRW. FSW and HRW living with HIV in turn increased the risk of HIV infection for other clients of FSW, MWUD, and their regular non-key population male partners. These factors in combination strongly support stakeholder recommendations for low-cost interventions to make HIV testing services available to accessible populations of MWUD who are accessing other support services, which will also improve the quality of future data around these overlapping populations.

New HIV infections through male-to-male sexual transmission were estimated to be low, but these key populations remained at disproportionate risk due to lower access to HIV services, experiencing stigma^10^^,^^23^ and having multiple sex partners.^21^ Although MSM and TGW were estimated to contribute minimally to new infections over time, previous studies of regional trends in HIV transmission risk for these groups suggest an increased burden of HIV infections among MSM and TGW.^41^^,^^42^ Any introduced HIV infections could rapidly change the epidemic among MSM and TGW in Bhutan, so these populations require continued HIV prevention programming tailored to local needs.

Vertical transmission rates remained low due to the integrated component of prevention of mother-to-child transmission (PMTCT) services in the antenatal care system. Lower modelled vertical transmission reflects both that a higher proportion of people infected through vertical transmission are likely to have been diagnosed, and that vertical transmission rates in recent years (under-represented in the survey) are likely to have dropped steeply due to the national implementation of interventions to prevent vertical transmission in Bhutan. Despite low incidence rates of MTCT, antenatal testing and PMTCT remains an important part of the national response towards the triple elimination of MTCT of HIV, syphilis and hepatitis.^3^

The findings from this analysis may not be generalizable to other HIV epidemics in low prevalence settings. For example, within the same region Bangladesh and Nepal are also considered as having an overall low HIV prevalence, but research relating to the HIV epidemics in these countries suggest that it is largely concentrated among PWID.^43^^,^^44^ Moreover, the inclusion of MWUD modelled as a single population group in Bhutan may not be applicable in settings with a low prevalence of alcohol consumption or alcohol use disorders.

There were several limitations in the current study. Firstly, the epidemiological and behavioural data were collated from population surveys and programmatic data that may have varying degrees and types of biases. Secondly, the small population size of key populations, the low-level epidemic and the low HIV programmatic coverage led to uncertainty in accurately estimating the true HIV prevalence, as highlighted by the wide uncertainty intervals of EPP-Spectrum HIV estimates. This uncertainty would likely reflect differences outside of accessible key populations, where prevalence surveys have been conducted. Thus, if national HIV prevalence were found to be at the higher end of EPP-Spectrum HIV estimates, this could imply that there is greater risk among populations what were not included in the 2021 HSS study, such as among MWUD, including unidentified PWID. If national HIV prevalence were found to be at the lower end of EPP-Spectrum HIV estimates this would likely indicate lower risk transitions out of key populations, implying lower incidence relating to sex work and lower HIV prevalence among non-key populations.

However, based on the best available national evidence, the results provided novel understanding of the pathways of HIV transmission highlighting the need for enhanced efforts targeting the most affected groups. With this low-level epidemic, there is both risk and opportunity that the trajectory could change rapidly based on additional infections, or diagnoses and treatment within a year. Finally, data inputs were primarily undertaken from surveys pre-COVID-19 and during the early stage of COVID-19 based on program data from 2020, which may not accurately reflect long-term changes in risk. Therefore, future studies should be conducted to assess the impact of COVID-19 on HIV transmission risks and the national HIV epidemic as data becomes available.

Our findings highlight the burden of HIV associated with high-risk sex contacts among FSW, clients of FSW, and HRW. The complexity of relationships and transitions between key population and non-key population groups implied that greater effort should be made to increase coverage of HIV prevention and testing, and to reduce barriers to access to HIV services. Focusing future research and programmatic efforts on developing strategies that can prevent new HIV infections among individuals currently at risk and identify undiagnosed HIV infections among those with historic risk and their partners could help achieve the effective elimination of HIV transmission in Bhutan.

## Contributors

NW, LK, and RMH conceptualized the study. NW, LK, GS, TD and RMH collated the data. NW and RMH led the technical analysis. NW took the lead in manuscript writing and revisions. LK and RMH provided overall direction and planning for the study. LK, DtB, GS, TD, YYS, AB, NS, KB, RMH contributed to the interpretation of findings, and reviewed and edited the manuscript.

## Data sharing statement

The Optima HIV model is free and open source, and is available from GitHub https://github.com/optimamodel/optima with a user-interface accessible from https://optimamodel.com/hiv/.

Data used to inform this analysis is available in the report available from https://www.burnet.edu.au/knowledge-and-media/research-reports-plus-policy-briefs/analyses-for-impact-efficiency-and-sustainability-of-priority-key-population-hiv-services-in-asia-bhutan-2023/. Proposals to access original country data owned by National HIV/AIDS Control Programme Bhutan should be directed to the authors and will require a formal data access agreement. Given the study's setting and sample size, even an anonymized dataset poses a significant risk of identifying a participant. Such data can inadvertently reveal a participant's identity or be perceived as compromising their anonymity. Due to these concerns, releasing primary data publicly is not deemed safe in many instances. However, requests for data access will be carefully evaluated on a case-by-case basis to ensure participant confidentiality is maintained.

## Declaration of interests

The authors declare no competing interests.
