# Supplementary material for: Establishing HIV transmission pathways in Bhutan: a modelling study
Source: Lancet Reg Health Southeast Asia. 2025 Sep 29;42:100676. doi: 10.1016/j.lansea.2025.100676 (PMC12513292; doi:10.1016/j.lansea.2025.100676)
Supplement: Appendix [file mmc1.docx]

**Supplementary material**

Supplementary material appendices to:

Establishing HIV transmission pathways in Bhutan: a modelling study.

Table of Contents

[Appendix A. Stakeholder discussion 2](#_Toc207727037)

[Table A 1. List of stakeholders and key topics discussed during consultation. 2](#_Toc207727038)

[Appendix B. Technical summary of the Optima HIV model 4](#_Toc207727039)

[Methodology for calibration 4](#_Toc207727040)

[Model parameters 4](#_Toc207727041)

[Table B 1. Model parameters: transmissibility, disease progression and disutility weights. 4](#_Toc207727042)

[Table B 2. Model parameters: treatment recovery and CD4 changes due to ART, and death rates. 5](#_Toc207727043)

[Figure B 1. Optima HIV model structure. 6](#_Toc207727044)

[Model inputs 6](#_Toc207727045)

[Table B 3. Model inputs and data sources. 7](#_Toc207727046)

[Appendix C. Additional results 10](#_Toc207727047)

[Table C 1. Estimated number of annual new HIV infections with uncertainty ranges from 1990 to 2022. 10](#_Toc207727048)

[Table C 2. Estimated number of annual people living with HIV with uncertainty ranges from 1990 to 2022. 11](#_Toc207727049)

[Table C 3. Estimated number of annual HIV-related deaths with uncertainty ranges from 1990 to 2022. 12](#_Toc207727050)

[References 13](#_Toc207727051)

1. Stakeholder discussion

Stakeholder discussions were conducted throughout January to June 2022 with individual organizations and technical working group.

Table A 1. List of stakeholders and key topics discussed during consultation.

| **Stakeholder** | **Key discussion points** |
| --- | --- |
| The Global Fund to Fight AIDS, Tuberculosis and Malaria, Ministry of Health | - HIV funding landscape; and prioritization of funds for key population programs. |
| Multi-Sector Task Force, MoH | - Health awareness programs; and barriers in expanding HIV prevention-related programs. |
| Policy and Planning Division, MoH | - HIV testing, monitoring, reporting systems. |
| Care, Support and Treatment Unit | - HIV treatment, care and support pathways, ART drug regimen; adherence programs; impact of COVID-19 pandemic on ART; and training for service providers. |
| Health Information Service Centre | - HIV testing and treatment programs; outreach activities; and barriers in accessing HIV services. |
| Bhutan Narcotics Control Authority | - Review estimates for the number of people who use alcohol and other drugs, and risk-taking behaviour among people who use alcohol and other drugs; and treatment and rehabilitation programs for people who use alcohol and other drugs. |
| Lhak-Sam | - HIV prevention programs; stigma and discrimination towards people living with HIV; and potential of income generation programs. |
| Pride Bhutan | - Population size estimation of transgender people; and HIV prevention services for transgender people. |
| Red Purse Network | - Population size estimation of female sex workers and other high-risk women; working duration of female sex workers and other high-risk women; HIV prevention services for female sex workers and other high-risk women; outreach costs for female sex workers and other high-risk women; and implications of drayang closures on reaching female sex workers and other high-risk women. |
| Chithuen Phendey Association | - Risk-taking behaviour among people who use alcohol and other drugs/people who inject drugs; prevention programs and rehabilitation services for people who use alcohol and other drugs; and barriers in increasing the program coverage. |
| **Technical working group**  Membership   - National HIV/AIDS Control Programme, MoH - Policy & Planning Division, MoH - Directorate Services, MoH - Care Support and Treatment Unit, Jigme Dorji Wangchuck National Referral Hospital - Health Information Service Centre - Lhak-Sam - Pride Bhutan - Chithuen Phendey Association - Save the Children Bhutan   With participation from:   - Department of Public Health - Country Coordinating Mechanism, Global Fund - Noncommunicable Disease Division, MoH - Health Promotion Division, MoH - Gross National Happiness Commission - Bhutan Narcotics Control Authority - Bhutan Health Trust Fund - Ministry of Finance - Red Purse Network - WHO - UNICEF - UNFPA - Burnet Institute - Health Equity Matters | Technical working group meetings focused on higher-level policy and planning questions relating to the wider allocative efficiency analysis conducted using the calibrated Optima HIV model. These meetings also included review, validation, and development of a shared understanding of model calibration and HIV transmission dynamics in Bhutan.  **Technical working group meeting #1** (March 2022)   - Introduction to Optima HIV modelling tool - Review of objectives of analysis and modelling process   **HIV stakeholder consultation workshop** (May 2022)  This workshop is to ensure that the allocative efficiency analysis responds to the right policy questions to inform high-level and long-term decision making for national financing of the HIV response given a constrained budget and other health priorities.   - Inform decision-makers about the rationale of allocative efficiency analysis - Explore policies limitations for HIV programming to key populations and parameters that will be used in the analysis - Understand the funding landscape and constraints around HIV response - Review realistic scenarios which should be tested by the analysis - Foster ownership of the project and promote the use of model outcomes for strategic planning   **Technical working group meeting #2** (June 2022)   - Validation of updated model calibration and results - Review of interpretation of results relating to key policy questions   **Dissemination of findings and recommendations**  **from HIV allocative efficiency analysis** (September 2022)  Presentation of modelling results (Burnet Institute)   - Pathways to HIV infection in Bhutan - Results of the allocative efficiency analysis - Policy implications to reach 2030 HIV elimination targets   Future planning (National HIV/AIDS Control Programme)   - Review of the domestic and international funding landscape and commitments toward HIV funding sustainability in Bhutan - Review the content and wording of the final policy brief - Feasibility and timeline to implement policy recommendations in line with HIV elimination targets |

ART, antiretroviral treatment; MoH, Ministry of Health; UNICEF, United Nations Children’s Fund; UNFPA, United Nations Population Fund; WHO, World Health Organization.

1. Technical summary of the Optima HIV model

This model is informed by the latest evidence on HIV transmission, disease progression, and the impact of HIV interventions on both. Table B 1 and Table B 2 list all model assumptions with associated references found in the [Optima HIV Vol. VI. Parameter Data Sources](https://docs.google.com/document/d/1VIxB08GjnLhUjRwLAKuBJ-To2WXud7krK9CNNu6NwIg/edit#heading=h.gjdgxs).

Optima HIV is s a compartmental HIV epidemic model which divides the entire population into compartments that (a) characterize their risk of transmitting a pathogen associated with disease, and/or (b) characterize their chance of experiencing morbidity or mortality (1). Movement between compartments is determined by the rates of transition.

The risks of transmitting, acquiring, and dying from HIV depend on a host of different factors that can vary across the population, across partnerships, and over time. In the Optima HIV epidemic model, the population is stratified in three different ways to reflect this variation: by demographic and/or risk group, by health/disease state (stratified by CD4 count category), and by stage of care. Optima HIV defines the different demographic/risk groups as populations, the different disease progression stages as health states, and the different care and treatment stages as care states. For example, a given person might be a female entertainment worker (their population) and be living with HIV with a CD4 count of 350–500 (their health state), and currently be linked to care but not on treatment (their care state).

To perform the optimization, Optima HIV uses a global parameter search algorithm called adaptive stochastic descent (ASD) (2). Optima HIV version 2.11.4 updated February 2023, available at <https://optimamodel.com/hiv/> was used for this analysis.

Methodology for calibration

The aim of calibration is to align model outputs to available epidemiological data and official country estimates based on other models (e.g. Spectrum) as best as possible given the underlying model structure and assumptions. The main calibration parameters used for Optima HIV are ‘initial prevalence’ (the percentage of each population with HIV in the first-time step of the model, January 1, 1990), and ‘force of infection’ which represents all factors which are not modelled explicitly but which impact on the likelihood of each population becoming infected relative to other populations. Calibration of parameters aims to align individual population prevalence estimates with prevalence survey data relating to each population, and secondarily to match existing country estimates including new HIV infections and HIV-related deaths from Spectrum modelling to provide consistency with an agreed baseline.

Model parameters

Three different types of HIV transmission are modelled: transmission between sexual partners, transmission via sharing injecting equipment, and mother-to-child transmission.

Table B 1. Model parameters: transmissibility, disease progression and disutility weights.

| Interaction-related transmissibility (% per act) | | |
| --- | --- | --- |
|  | Insertive penile-vaginal intercourse | 0.04% (0.01% - 0.14%) |
|  | Receptive penile-vaginal intercourse | 0.08% (0.06%-0.11%) |
|  | Insertive penile-anal intercourse | 0.11% (0.04%-0.28%) |
|  | Receptive penile-anal intercourse | 1.38% (1.02%-1.86%) |
|  | Intravenous injection | 0.80% (0.63%-2.40%) |
|  | Mother-to-child (breastfeeding) | 36.70% (29.40%-44.00%) |
|  | Mother-to-child (non-breastfeeding) | 20.50% (14.00%-27.00%) |
| Relative disease-related transmissibility | | |
|  | Acute infection | 5.60 (3.30-9.10) |
|  | CD4 (>500) | 1.00 (1.00-1.00) |
|  | CD4 (500) to CD4 (350-500) | 1.00 (1.00-1.00) |
|  | CD4 (200-350) | 1.00 (1.00-1.00) |
|  | CD4 (50-200) | 3.49 (1.76-6.92) |
|  | CD4 (<50) | 7.17 (3.90-12.08) |
| Disease progression (average years to move between disease progression stages) | | |
|  | Acute to CD4 (>500) | 0.24 (0.10-0.50) |
|  | CD4 (500) to CD4 (350-500) | 0.95 (0.62-1.16) |
|  | CD4 (350-500) to CD4 (200-350) | 3.00 (2.83-3.16) |
|  | CD4 (200-350) to CD4 (50-200) | 3.74 (3.48-4.00) |
|  | CD4 (50-200) to CD4 (<50) | 1.50 (1.13-2.25) |
| Changes in transmissibility (%) | | |
|  | Condom use | 95% (80%-98%) |
|  | Circumcision | 58% (47%-67%) |
|  | Diagnosis behaviour change | 0% (0%-68%) |
|  | STI cofactor increase | 265% (135%-519%) |
|  | Opioid substitution therapy | 54% (33%-68%) |
|  | PMTCT | 90% (82%-93%) |
|  | ARV-based pre-exposure prophylaxis | 95% (92%-97%) |
|  | ARV-based post-exposure prophylaxis | 73% (65%-80%) |
|  | ART not achieving viral suppression | 50% (30%-80%) |
|  | ART achieving viral suppression | 100% (92%-100%) |
| Disutility weights | | |
|  | Untreated HIV, acute | 0.18 (0.05-0.21) |
|  | Untreated HIV, CD4 (>500) | 0.01 (0.01-0.01) |
|  | Untreated HIV, CD4 (350-500) | 0.03 (0.01-0.04) |
|  | Untreated HIV, CD4 (200-350) | 0.08 (0.05-0.09) |
|  | Untreated HIV, CD4 (50-200) | 0.29 (0.11-0.47) |
|  | Untreated HIV, CD4 (<50) | 0.58 (0.38-0.72) |
|  | Treated HIV | 0.08 (0.03-0.11) |

Source: [Optima HIV User Guide Volume VI Parameter Data Sources](https://docs.google.com/document/d/1VIxB08GjnLhUjRwLAKuBJ-To2WXud7krK9CNNu6NwIg/edit#heading=h.gjdgxs).

Table B 2. Model parameters: treatment recovery and CD4 changes due to ART, and death rates.

| Treatment recovery due to suppressive ART (average years to move between disease progression stages) | | |
| --- | --- | --- |
|  | CD4 (350-500) to CD4 (>500) | 2.20 (1.07-7.28) |
|  | CD4 (200-350) to CD4 (350-500) | 1.42 (0.90-3.42) |
|  | CD4 (50-200) to CD4 (200-350) | 2.14 (1.39-3.58) |
|  | CD4 (<50) to CD4 (50-200) | 0.66 (0.51-0.94) |
|  | Time after initiating ART to achieve viral suppression (year) | 0.20 (0.10-0.30) |
| CD4 change due to non-suppressive ART (%/year) | | |
|  | CD4 (500) to CD4 (350-500) | 2.6% (0.5%-27.5%) |
|  | CD4 (350-500) to CD4 (>500) | 15.0% (3.8%-88.5%) |
|  | CD4 (350-500) to CD4 (200-350) | 10.0% (2.2%-87.0%) |
|  | CD4 (200-350) to CD4 (350-500) | 5.3% (0.8%-82.7%) |
|  | CD4 (200-350) to CD4 (50-200) | 16.2% (5.0%-86.9%) |
|  | CD4 (50-200) to CD4 (200-350) | 11.7% (3.2%-68.6%) |
|  | CD4 (50-200) to CD4 (<50) | 9.0% (1.9%_72.3%) |
|  | CD4 (<50) to CD4 (50-200) | 11.1% (4.7%-56.3%) |
| Death rate (% HIV-related mortality per year) | | |
|  | Acute infection | 0.36% (0.29%-0.44%) |
|  | CD4 (>500) | 0.36% (0.29%-0.44%) |
|  | CD4 (350-500) | 0.58% (0.48%-0.71%) |
|  | CD4 (200-350) | 0.88% (0.75%-1.01%) |
|  | CD4 (50-200) | 5.90% (5.40%-7.90%) |
|  | CD4 (<50) | 32.00% (29.60%-43.20%) |
|  | Relative death rate on ART achieving viral suppression | 23.00% (15.00%-30.00%) |
|  | Relative death rate on ART not achieving viral suppression | 49.00% (28.35%-84.17%) |
|  | Tuberculosis factor | 217% (127%-371%) |

Source: [Optima HIV User Guide Volume VI Parameter Data Sources](https://docs.google.com/document/d/1VIxB08GjnLhUjRwLAKuBJ-To2WXud7krK9CNNu6NwIg/edit#heading=h.gjdgxs).

Optima HIV models seven states related to the care and treatment cascade (susceptible, undiagnosed, diagnosed and never linked to care, in care and not receiving ART, receiving ART and not achieved virally suppression, receiving ART and achieved virally suppression, and lost-to-follow-up). Among male populations, the susceptible compartment is further divided into those who have been circumcised versus those who have not been circumcised. All infected stages are further disaggregated into six CD4-related health states. Taken together, this gives 38 health and care states (Figure B 1; circumcised compartments modelled for male populations only and not shown).

Figure B 1. Optima HIV model structure.


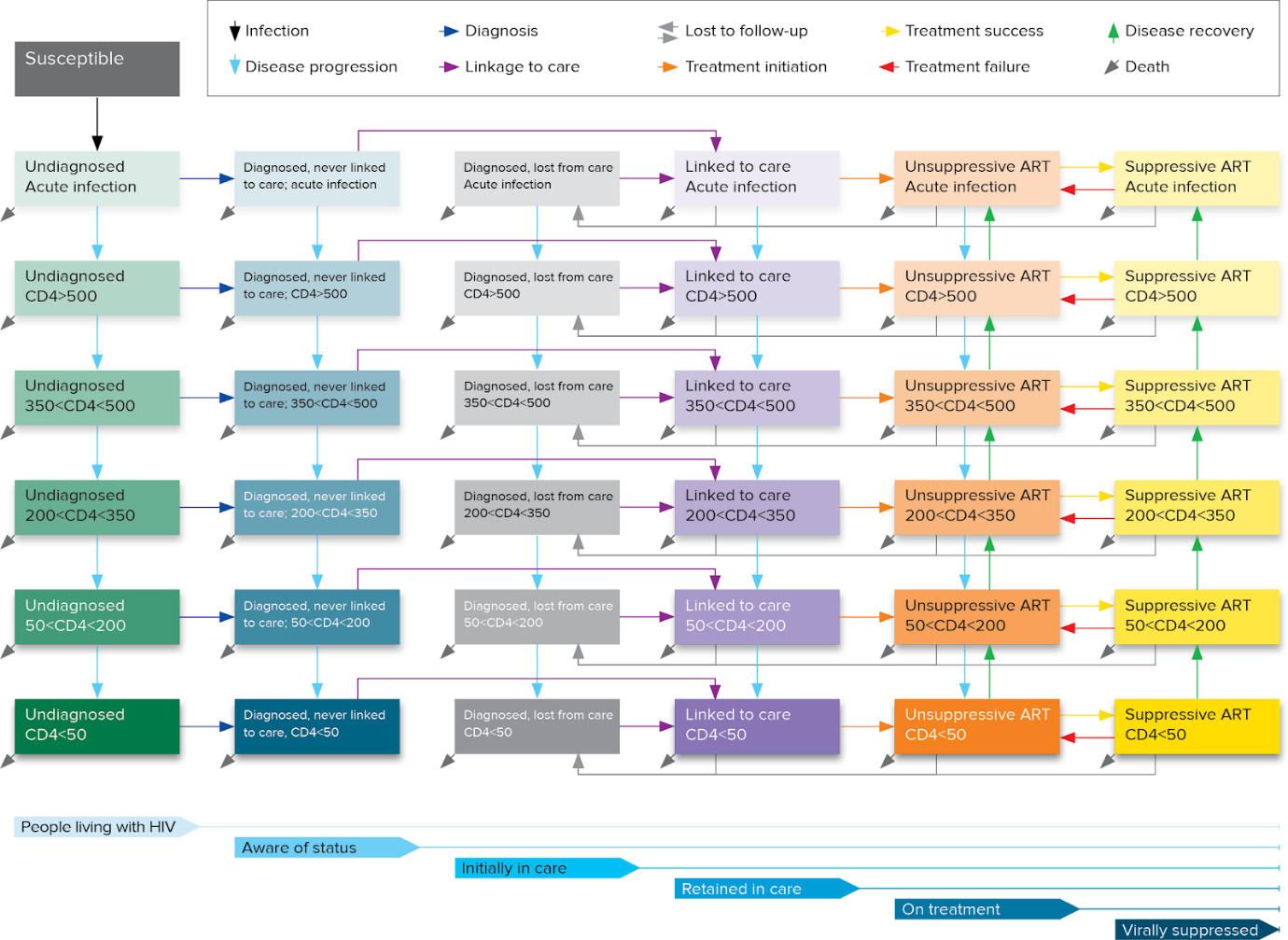


Model inputs

Epidemiological, behavioural and programmatic data informing the Optima HIV model for participating countries were sourced from national records, integrated biological-behavioural surveillance (IBBS) surveys, household surveys and other studies supplemented by expert advice from stakeholder consultations. The input data associated with populations, testing, treatment and cascade, sexual partnerships, injecting behaviours, and partnerships are outlined in Table B 3.

Table B 3. Model inputs and data sources.

| **Parameter** | Source |
| --- | --- |
| Population size^*^ | Age and gender stratified population sizes from the United Nations World Population Prospects 2019 (3).  Key population sizes for higher risk populations are estimated from various sources (4, 5). |
| HIV prevalence by population groups^*^ | HIV prevalence data values are used as the primary point of reference during calibration. Values are taken from a combination of primary research including survey data, where available, and expert opinion/assumptions where no data exists (6-9). |
| Other epidemiology^*^   - Percentage of people who die from non-HIV-related causes per year - Prevalence of any ulcerative STIs - Tuberculosis prevalence | Background mortality(3) and supplementary comorbidity information is taken from various sources (5, 8, 10-16). |
| Testing and treatment^*^   - Percentage of population tested for HIV in the last 12 months - Probability of a person with CD4<200 being tested per year - Number of people on treatment - Proportion of exposure events covered by ARV-based pre-exposure prophylaxis - Proportion of exposure events covered by ARV-based post-exposure prophylaxis - Number of women on PMTCT (Option B/B+) - Birth rate (births per woman per year) - Percentage of HIV-positive women who breastfeed | The percentage of the population tested per year represents the likelihood that someone with an undiagnosed HIV infection will be diagnosed over the course of a year. As such inputs may be adjusted as part of calibration to match the proportion of HIV infections estimated to be diagnosed in each year, while maintaining trends in reported testing percentages. Data inputs for testing and treatment are taken from various sources (5, 8, 9, 17-20). |
| Optional indicators^*^   - Number of HIV tests per year - Number of HIV diagnoses per year - Modelled estimate of new HIV infections per year - Modelled estimate of HIV prevalence - Modelled estimate of number of PLHIV - Number of HIV-related deaths - Number of people initiating ART each year - PLHIV aware of their status (%) - Diagnosed PLHIV in care (%) - PLHIV in care on treatment (%) - Pregnant women on PMTCT (%) - People on ART with viral suppression (%) | Data entered in this section of the Optima HIV databook is not used by the model directly to generate output, but rather allows comparison points to be entered from other reliable sources or models in order to ensure consistency. Optional indicators data are compiled through various sources (9, 17, 21-25). |
| Cascade^*^   - Average time taken to be linked to care (years) (by population groups) - Average time taken to be linked to care for people with CD4<200 (years) - Percentage of people in care who are lost to follow-up per year (%/year) - Percentage of people with CD4<200 lost to follow up (%/year) - Percentage of people lost to follow-up who are returned to care per year (%/year) - Viral load monitoring (number/year) - Proportion of those with identified viral load failure who are provided with effective adherence support or a successful new regimen (%/year) - Treatment failure rate | Cascade parameters informed by programmatic data (21, 26). |
| Sexual behaviour^*^   - Average number of acts with regular partners per person per year - Average number of acts with casual partners per person per year - Average number of acts with transactional partners per person per year - % age of people who used a condom at last act with regular partners - Percentage of people who used a condom at last act with casual partners - Percentage of people who used a condom at last act with transactional partners - Percentage of males who have been traditionally circumcised - Number of voluntary medical male circumcisions | Sources for sexual behaviour(5, 7, 8, 10, 11, 27) and programmatic data, with circumcision estimate was informed through global prevalence of male circumcision (28). |
| Injecting behaviours^*^   - Average number of injections per person per year - Percentage of people who receptively shared a needle/syringe at last injection - Number of people who inject drugs who are on opiate substitution therapy (OST) | Sources for injecting behaviour informed by the expert advice. |
| Partnerships and transitions   - Interactions between regular partners - Interactions between casual partners - Interactions between transactional partners - Interactions between people who inject drugs - Birth - Aging - Risk-related population transitions (average number of years before movement) | Informed by population definitions, supplemented by details from multiple sources (5, 7). |
| Migration^*^   - Percentage of people who emigrate per year - Number of people who immigrate into population per year - HIV prevalence of immigrants into population per year   Proportion of people living with HIV who immigrate who are diagnosed prior to arrival | Optionally included depending on significance to epidemic and available data. Migration is not modelled in the Bhutan analysis. |
| Constants   - Interaction-related transmissibility (% per act) - Relative disease-related transmissibility - Disease progression (average years to move between disease progression stages) - Treatment recovery due to suppressive ART (average years to move between disease progression stages) - CD4 change due to non-suppressive ART (%/year) - Death rate (% mortality per year) - Changes in transmissibility (%) - Disutility weights | Sources for constant values used for Optima HIV are given in the Optima HIV user guide (29). |

*Values can be defined annually from 1990 to 2022.

ART, antiretroviral treatment; IBBS, integrated biological behavioural surveillance surveys; PLHIV, people living with HIV; PMTCT, prevention of mother-to-child transmission (vertical transmission); STI, sexually transmitted infection.

1. Additional results

Table C 1. Estimated number of annual new HIV infections with uncertainty ranges from 1990 to 2022.

|  | Point estimate | Lower bound | Higher bound |
| --- | --- | --- | --- |
| 1990 | 8 | 7 | 8 |
| 1991 | 12 | 12 | 13 |
| 1992 | 14 | 14 | 15 |
| 1993 | 17 | 16 | 18 |
| 1994 | 20 | 19 | 21 |
| 1995 | 28 | 26 | 30 |
| 1996 | 38 | 35 | 41 |
| 1997 | 50 | 46 | 54 |
| 1998 | 68 | 62 | 75 |
| 1999 | 85 | 77 | 94 |
| 2000 | 101 | 92 | 114 |
| 2001 | 111 | 99 | 124 |
| 2002 | 120 | 107 | 135 |
| 2003 | 130 | 115 | 146 |
| 2004 | 134 | 119 | 151 |
| 2005 | 137 | 120 | 154 |
| 2006 | 131 | 115 | 148 |
| 2007 | 126 | 110 | 143 |
| 2008 | 115 | 100 | 132 |
| 2009 | 107 | 93 | 123 |
| 2010 | 102 | 88 | 117 |
| 2011 | 99 | 85 | 113 |
| 2012 | 96 | 82 | 110 |
| 2013 | 94 | 80 | 108 |
| 2014 | 90 | 77 | 104 |
| 2015 | 87 | 74 | 100 |
| 2016 | 84 | 71 | 98 |
| 2017 | 82 | 67 | 97 |
| 2018 | 79 | 64 | 95 |
| 2019 | 75 | 59 | 92 |
| 2020 | 69 | 54 | 87 |
| 2021 | 66 | 51 | 84 |
| 2022 | 64 | 48 | 82 |

Table C 2. Estimated number of annual people living with HIV with uncertainty ranges from 1990 to 2022.

|  | Point estimate | Lower bound | Higher bound |
| --- | --- | --- | --- |
| 1990 | 17 | 16 | 17 |
| 1991 | 25 | 24 | 26 |
| 1992 | 36 | 35 | 38 |
| 1993 | 50 | 48 | 52 |
| 1994 | 65 | 63 | 69 |
| 1995 | 85 | 81 | 90 |
| 1996 | 114 | 109 | 121 |
| 1997 | 152 | 144 | 163 |
| 1998 | 203 | 191 | 219 |
| 1999 | 270 | 252 | 294 |
| 2000 | 352 | 324 | 384 |
| 2001 | 445 | 407 | 488 |
| 2002 | 544 | 495 | 599 |
| 2003 | 647 | 587 | 717 |
| 2004 | 753 | 681 | 839 |
| 2005 | 856 | 772 | 960 |
| 2006 | 953 | 856 | 1,074 |
| 2007 | 1,037 | 926 | 1,172 |
| 2008 | 1,107 | 983 | 1,252 |
| 2009 | 1,160 | 1,025 | 1,314 |
| 2010 | 1,200 | 1,057 | 1,362 |
| 2011 | 1,230 | 1,082 | 1,399 |
| 2012 | 1,252 | 1,100 | 1,428 |
| 2013 | 1,269 | 1,113 | 1,447 |
| 2014 | 1,280 | 1,120 | 1,458 |
| 2015 | 1,285 | 1,122 | 1,466 |
| 2016 | 1,288 | 1,124 | 1,474 |
| 2017 | 1,293 | 1,126 | 1,486 |
| 2018 | 1,302 | 1,132 | 1,497 |
| 2019 | 1,312 | 1,138 | 1,508 |
| 2020 | 1,321 | 1,143 | 1.518 |
| 2021 | 1,328 | 1,147 | 1,526 |
| 2022 | 1,333 | 1,151 | 1,535 |

Table C 3. Estimated number of annual HIV-related deaths with uncertainty ranges from 1990 to 2022.

|  | Point estimate | Lower bound | Higher bound |
| --- | --- | --- | --- |
| 1990 | 2 | 1 | 2 |
| 1991 | 2 | 1 | 2 |
| 1992 | 2 | 1 | 2 |
| 1993 | 2 | 2 | 2 |
| 1994 | 2 | 2 | 2 |
| 1995 | 2 | 2 | 2 |
| 1996 | 3 | 3 | 3 |
| 1997 | 4 | 4 | 4 |
| 1998 | 5 | 5 | 5 |
| 1999 | 7 | 6 | 7 |
| 2000 | 9 | 8 | 10 |
| 2001 | 12 | 11 | 13 |
| 2002 | 15 | 14 | 17 |
| 2003 | 20 | 18 | 22 |
| 2004 | 25 | 23 | 28 |
| 2005 | 31 | 28 | 35 |
| 2006 | 37 | 33 | 42 |
| 2007 | 44 | 39 | 50 |
| 2008 | 51 | 45 | 58 |
| 2009 | 57 | 51 | 66 |
| 2010 | 63 | 55 | 73 |
| 2011 | 68 | 59 | 80 |
| 2012 | 72 | 62 | 84 |
| 2013 | 76 | 65 | 89 |
| 2014 | 79 | 68 | 93 |
| 2015 | 79 | 67 | 93 |
| 2016 | 75 | 63 | 89 |
| 2017 | 65 | 57 | 83 |
| 2018 | 64 | 51 | 78 |
| 2019 | 58 | 46 | 73 |
| 2020 | 55 | 43 | 69 |
| 2021 | 53 | 41 | 67 |
| 2022 | 51 | 39 | 65 |

# References

1. Kerr CC, Stuart RM, Gray RT, Shattock AJ, Fraser-Hurt N, Benedikt C, et al. Optima: A Model for HIV Epidemic Analysis, Program Prioritization, and Resource Optimization. J Acquir Immune Defic Syndr. 2015;69(3):365-76.

2. Kerr CC, Dura-Bernal S, Smolinski TG, Chadderdon GL, Wilson DP. Optimization by Adaptive Stochastic Descent. PLoS One. 2018;13(3):e0192944.

3. United Nations Department of Economic and Social Affairs/Population Division. World Population Prospects 2019 (online edition). 2019.

4. Bhutan Narcotics Control Agency, United Nations Office on Drugs and Crime. National baseline assessment of drugs and controlled substance use in Bhutan. 2009.

5. National AIDS Control Programme. Mapping and population size estimation of men who have sex with men, transgender persons and high-risk women in Bhutan. 2020.

6. National AIDS Control Programme. HIV Sentinel Surveillance among Key Populations in Bhutan. 2021.

7. Khandu L, Zwanikken PAC, Wangdi S. HIV Vulnerability and Sexual Risk Behaviour of the Drayang Girls in Bhutan. SAARC Journal of Tuberculosis, Lung Diseases and HIV/AIDS. 2019;17(1).

8. School of Planning Monitoring Evaluation and Research. Integrated biological and behavioral surveillance (IBBS) among vulnerable and key populations at higher risk in Bhutan, 2016. 2016.

9. Ministry of Health of the Royal Government of Bhutan. Progress report of the Global Fund disbursement. 2021.

10. Department of Youth and Sports, Ministry of Education. An assessment of vulnerable and at-risk adolescents (13-18 years) in Bhutan: exploring social and health risk behaviours. 2009.

11. Ministry of Health of the Royal Government of Bhutan. HIV/AIDS behaviour survey among the general population in Bhutan, 2006. 2008.

12. United Nations Department of Economic and Social Affairs/Population Division. World Population Prospects 2019: Male deaths by five-year age group, region, subregion and country (online edition). 2019.

13. United Nations Department of Economic and Social Affairs/Population Division. World Population Prospects 2019: Female deaths by five-year age group, region, subregion and country (online edition). 2019.

14. United Nations Department of Economic and Social Affairs/Population Division. World Population Prospects 2019: Female population by five-year age group, region, subregion and country (online edition). 2019.

15. United Nations Department of Economic and Social Affairs/Population Division. World Population Prospects 2019: Male population by five-year age group, region, subregion and country (online edition). 2019.

16. United Nations Department of Economic and Social Affairs/Population Division. World Population Prospects 2019: Probabilistic projection of total population by five-year age group, region, subregion and country (online edition). 2019.

17. UNAIDS Spectrum. Spectrum for HIV projection in Bhutan. 2022.

18. National Statistics Bureau. Bhutan multiple indicator survey. Thimphu, Bhutan; 2011.

19. United Nations Department of Economic and Social Affairs/Population Division. World Population Prospects 2019: Age-specific fertility rates by region, subregion and country (online edition). 2019.

20. United Nations Department of Economic and Social Affairs/Population Division. World Population Prospects 2019: Infant mortality rate by region, subregion and country (online edition). 2019.

21. Ministry of Health of the Royal Government of Bhutan. Annual health bulletin 2021. Thimphu, Bhutan: Policy and Planning Division, Ministry of Health; 2021.

22. National HIV/AIDS, Hepatitis and STIs Control Programme. Retrospective risk assessment of current living HIV cases diagnosed from 1993-2020. Thimphu: Ministry of Health, Royal Government of Bhutan; 2021.

23. National AIDS Control Programme. Review of the package of HIV services for key populations in Bhutan. 2021.

24. Ministry of Health of the Royal Government of Bhutan. Progress report of the Global Fund disbursement. 2019.

25. Global AIDS Monitoring. Bhutan country progress report. 2020.

26. Ministry of Health of the Royal Government of Bhutan. Annual health bulletin 2020. Thimphu, Bhutan: Policy and Planning Division, Ministry of Health; 2020.

27. Norbu K, Mukhia S, Tshokey. Assessment of knowledge on sexually transmitted infections and sexual risk behaviour in two rural districts of Bhutan. BMC Public Health. 2013;13(1):1142.

28. Morris BJ, Wamai RG, Henebeng EB, Tobian AAR, Klausner JD, Banerjee J, et al. Estimation of country-specific and global prevalence of male circumcision. Population Health Metrics. 2016;14(1):4.

29. Optima Consortium for Decision Science. Optima HIV User Guide, Volume VI: Parameter Data Sources Optima Consortium for Decision Science 2021 [Available from: <https://docs.google.com/document/d/1VIxB08GjnLhUjRwLAKuBJ-To2WXud7krK9CNNu6NwIg/edit?usp=sharing>.
